# Draft Genome Sequences of Four *Citrobacter* Isolates Recovered from Wild Australian Shorebirds

**DOI:** 10.1128/MRA.01113-20

**Published:** 2021-01-07

**Authors:** Hannah G. P. Smith, David C. Bean, William Pitchers, Mary Valcanis, Rohan H. Clarke, Richard Loyn, Chris J. Hassell, Andrew R. Greenhill

**Affiliations:** a School of Science, Psychology and Sport, Federation University Australia, Ballarat, Victoria, Australia; b Microbiological Diagnostic Unit Public Health Laboratory, Department of Microbiology and Immunology, University of Melbourne at The Peter Doherty Institute for Infection and Immunity, Melbourne, Australia; c School of Biological Sciences, Monash University, Melbourne, Victoria, Australia; d Centre for Freshwater Ecosystems, School of Life Sciences, La Trobe University, Melbourne, Victoria, Australia; e Eco Insights, Melbourne, Victoria, Australia; f Global Flyway Network, Broome, Western Australia, Australia; Loyola University Chicago

## Abstract

*Citrobacter* is a ubiquitous bacterial genus whose members inhabit a variety of niches. Some species are clinically important for both antimicrobial resistance (AMR) carriage and as the cause of nosocomial infections. Surveillance of *Citrobacter* species in the environment can provide indicators of the spread of AMR genes outside clinical spaces. In this study, we present draft genome sequences of four *Citrobacter* isolates obtained from three species of wild Australian shorebirds.

## ANNOUNCEMENT

The genus *Citrobacter* comprises 11 species. They occupy a broad range of habitats, play a key role in the nitrogen cycle, and are frequently found in food and in the gut of animals, including humans (1). *Citrobacter* is an opportunistic pathogen of humans, most commonly associated with infant meningitis, urinary tract infections, sepsis, and pneumonia (2). The species most commonly isolated from clinical specimens are C. koseri, C. freundii, C. youngae, C. braakii, and C. amalonaticus (3). *Citrobacter* species can act as reservoirs for antimicrobial resistance (AMR) genes and can transfer these genes to other pathogenic bacteria (4, 5). *Citrobacter* spp. have been isolated from both healthy (6) and diseased birds (7).

We present here draft genomes of four *Citrobacter* isolates collected from Australian shorebirds through 2017 to 2018.

Four *Citrobacter* isolates were recovered from cloacal swabs collected from healthy Australian shorebirds (Table 1). The cloacal swabs were preenriched by incubating overnight in brain heart infusion broth at 35°C, followed by a secondary enrichment by transferring 100 µl into Mannitol broth and again incubating overnight at 35°C. The broths were subsequently subcultured onto MacConkey II agar (Oxoid) and incubated overnight at 35°C.

**TABLE 1:** Genotypic and phenotypic features of the *Citrobacter* sp. isolates

Characteristic	Data for strain:			
	966a	1120a	1241a	1273b
Species	Citrobacter amalonaticus	Citrobacter braakii	Citrobacter freundii	Citrobacter freundii
Sampling location	38.324105 S, 145.517553 E	38.003826 S, 144.596880 E	17.979327 S, 122.336533 E	17.979327 S, 122.336533 E
Host	Double-banded plover (Charadrius bicinctus)	Curlew sandpiper (Calidris ferruginea)	Bar-tailed godwit (Limosa lapponica)	Bar-tailed godwit (*Limosa lapponica*)
Yr of isolation	2017	2017	2018	2018
Phenotypic resistance	Amoxicillin, ampicillin	Amoxicillin, ampicillin	Amoxicillin	Amoxicillin, ampicillin
AMR^*a*^ gene	None	*bla*_CMY-48_	*bla*_CMY-48_	*bla*_CMY-48_
Total no. of raw paired-end reads	753,330	617,160	728,982	493,188
No. of contigs	91	34	48	54
Total length (bp)	4,903,911	5,098,689	5,280,444	5,279,325
*N*_50_ length (bp)	225,898	851,789	571,851	571,851
Avg depth (×)	71.7	71.7	71.7	68.5
GC content (%)	53.35	51.49	51.35	54
BioSample accession no.	SAMN13884683	SAMN13884692	SAMN13884697	SAMN13884710
Assembly accession no.	GCF_014333035.1	GCF_014332945.1	GCF_014332855.1	GCF_014332835.1

a AMR, antimicrobial resistance.

Phenotypic testing of antimicrobial resistance was conducted using the disk diffusion method (8). For DNA extraction, organisms were grown overnight on nutrient agar. Genomic DNA (gDNA) was extracted using a Qiagen DNeasy kit and quantified using an Invitrogen Qubit 2 fluorometer. Sequencing was conducted at the Australian Genome Research Facility using the Illumina MiSeq platform, with Illumina gDNA shotgun library preparation with the bead size selection protocol generating 150-bp paired-end reads.

The raw reads were uploaded to the Galaxy Web platform, and the data were analyzed via the public server at usegalaxy.org, version 20.01 (9). The genomes were assembled *de novo*, read quality control was performed using Unicycler version 0.4.8.0 (10), and the genome assembly quality was analyzed using QUAST version 5.0.2+galaxy1 (11). The genomes were uploaded to NCBI and annotated using PGAP version 4.12 (12). Further information on the genome parameters is given in Table 1.

ResFinder version 3.2 (13), hosted by the Centre for Genomic Epidemiology (http://www.genomicepidemiology.org/), was used to identify AMR genes. The AMR gene identified can be seen in Table 1. This gene is thought to have originated in C. freundii (14).

### Data availability.

The whole-genome sequences, assemblies, and raw reads for this project have been deposited in GenBank under the BioProject accession number PRJNA602163. Raw reads are available in the SRA under the accession numbers SRR11613026 (*C. braakii*), SRR11613020 (C. freundii), SRR11613006 (C. freundii), and SRR11612996 (*C. amalonaticus*).
